# Low pan-immune-inflammation-value predicts better chemotherapy response and survival in breast cancer patients treated with neoadjuvant chemotherapy

**DOI:** 10.1038/s41598-021-94184-7

**Published:** 2021-07-19

**Authors:** Ahmet Bilgehan Şahin, Erdem Cubukcu, Birol Ocak, Adem Deligonul, Sibel Oyucu Orhan, Sahsine Tolunay, Mustafa Sehsuvar Gokgoz, Sibel Cetintas, Gorkem Yarbas, Kazım Senol, Mehmet Refik Goktug, Zeki Burak Yanasma, Ulviyya Hasanzade, Turkkan Evrensel

**Affiliations:** 1 Department of Medical Oncology, School of Medicine, Bursa Uludag University, 16059 Gorukle, Nilufer, Bursa Turkey; 2 Department of Pathology, School of Medicine, Bursa Uludag University, Bursa, Turkey; 3 School of Medicine, General Surgery, Bursa Uludag University, Bursa, Turkey; 4 Department of Radiation Oncology, School of Medicine, Bursa Uludag University, Bursa, Turkey; 5 Department of Internal Medicine, School of Medicine, Bursa Uludag University, Bursa, Turkey

**Keywords:** Breast cancer, Cancer, Immunology, Oncology, Tumour immunology

## Abstract

Blood-based biomarkers reflect systemic inflammation status and have prognostic and predictive value in solid malignancies. As a recently defined biomarker, Pan-Immune-Inflammation-Value (PIV) integrates different peripheral blood cell subpopulations. This retrospective study of collected data aimed to assess whether PIV may predict the pathological complete response (pCR) to neoadjuvant chemotherapy (NAC) in Turkish women with breast cancer. The study consisted of 743 patients with breast cancer who were scheduled to undergo NAC before attempting cytoreductive surgery. A pre-treatment complete blood count was obtained in the two weeks preceding NAC, and blood-based biomarkers were calculated from absolute counts of relevant cell populations. The pCR was defined as the absence of tumor cells in both the mastectomy specimen and lymph nodes. Secondary outcome measures included disease-free survival (DFS) and overall survival (OS). One hundred seven patients (14.4%) had pCR. In receiver operating characteristic analysis, optimal cut-off values for the neutrophile-to-lymphocyte ratio (NLR), monocyte-to-lymphocyte ratio (MLR), platelet-to-lymphocyte (PLR), PIV, and Ki-67 index were determined as ≥ 2.34, ≥ 0.22, ≥ 131.8, ≥ 306.4, and  ≥ 27, respectively. The clinical tumor (T) stage, NLR, MLR, PLR, PIV, estrogen receptor (ER) status, human epidermal growth factor receptor-2 (HER-2) status, and Ki-67 index were significantly associated with NAC response in univariate analyses. However, multivariate analysis revealed that the clinical T stage, PIV, ER status, HER-2 status, and Ki-67 index were independent predictors for pCR. Moreover, the low PIV group patients had significantly better DFS and OS than those in the high PIV group (*p* = 0.034, *p* = 0.028, respectively). Based on our results, pre-treatment PIV seems as a predictor for pCR and survival, outperforming NLR, MLR, PLR in predicting pCR in Turkish women with breast cancer who received NAC. However, further studies are needed to confirm our findings.

## Introduction

Malignancies arising in the mammary gland are the most common type of cancer in women^1^. The lifetime risk of breast cancer for a woman has been calculated at around 1-in-7 to 1-in-10^2^, indicating that approximately 10% of the female population will be diagnosed with breast cancer during their lifetime^1,2^.

Neoadjuvant chemotherapy (NAC)—consisting of chemotherapy delivered before local treatment (surgery)—has become the standard of care in patients with locally advanced breast cancer^3^. In addition, downstaging of the primary tumor in patients with earlier stages of breast cancer may facilitate breast-conserving therapy and offer the opportunity to downstage the axilla—ultimately obviating the need for axillary treatment in some patients^4,5^. The overarching goal of NAC is to achieve pathological complete response (pCR)—which is, in turn, associated with a lower recurrence rate and more favorable survival outcomes^6^. Unfortunately, there is considerable interindividual variation in response to NAC amongst women with breast cancer, and several variables have been investigated in relation to this variability^7^.

In the last two decades, the relationship between chronic inflammation and cancer has become very popular, and both the diagnostic and therapeutic value of inflammatory markers have been studied extensively. Inflammation has been shown to promote tumor initiation and progression, whereas escape from immune surveillance may favor cancer invasiveness^8^. In the tumor microenvironment, neutrophils, monocytes-derived macrophages, and platelets have adverse prognostic significance by promoting tumoral angiogenesis and tumor growth, whereas tumor-infiltrating lymphocytes portend favorable outcomes^9–11^. Based on the assumption that peripheral blood cell populations can provide information about the intratumoral immune system status, peripheral blood-derived inflammation markers such as neutrophile-lymphocyte ratio (NLR), monocyte-lymphocyte ratio (MLR), and platelet-lymphocyte ratio (PLR) was shown to have prognostic value in many solid organ malignancies^12–14^. In addition to their prognostic value, these markers were reported to predict the neoadjuvant chemotherapy response in breast cancer^15–17^.

In 2020, Fuca et al. reported that a novel systemic immune score called Pan-Immune-Inflammation-Value (PIV) performed better in predicting survival outcomes than other immune-inflammatory biomarkers such as NLR in advanced colorectal cancer patients^18^. However, PIV's predictive and prognostic value in breast cancer patients receiving NAC has not been studied. We, therefore, designed the current study to address these issues specifically.

## Results

The general characteristics of the entire study sample (n = 743) are shown in Table 1. The median age was 48.0 years (range: 22.0–83.5 years). One hundred ninety-seven patients (26.5%) had T3/T4, and 37.5% had node-positive disease. More than two-thirds received chemotherapy regimens containing both anthracycline and taxane. Of patients, 14.4% had pCR.

**Table 1:** Clinicopathological characteristics of the patients.

Characteristic	N (743)	(%)
**Age**		
*Median (range), years*	48.0 (22.0–83.5)	
**Menopausal status**		
*Pre-menopausal*	379	(51.0)
*Post-menopausal*	364	(49.0)
**Clinical T stage**		
*T1-T2*	518	(69.7)
*T3-T4*	197	(26.5)
*Missing*	28	(3.8)
**Clinical node status**		
*Negative*	444	(59.8)
*Positive*	279	(37.5)
*Missing*	20	(2.7)
**Histotype**^a^		
*Invasive ductal carcinoma*	671	(90.3)
*Other histology types*^b^	53	(7.1)
*Missing*	19	(2.6)
**Estrogen receptor status**^a^		
*Positive*	484	(65.1)
*Negative*	184	(24.8)
*Missing*	75	(10.1)
**HER-2 status**^a^		
*Positive*	160	(21.5)
*Negative*	508	(68.4)
*Missing*	75	(10.1)
**Ki-67 index (%)**^a^		
*Median (range)*	26 (0–100)	
**Chemotherapy regimens**		
*Anthracycline plus taxane*	510	(68.6)
*Anthracycline-based regimens*	204	(27.5)
*Taxane-based regimens*	29	(3.9)
**pCR**		
*Yes*	107	(14.4)
*No*	636	(85.6)

*T* tumor, *pCR* pathological complete response, *HER-2* human epidermal growth factor receptor-2.

^a^Histopathological examination of the pre-treatment biopsy specimen.

^b^Including lobular, mucinous, papillary, metaplastic, and mixed tumors.

Table 2 presents the values of the area under the curve, sensitivity, and specificity in receiver operating characteristic (ROC) analysis. The cut-off values for the NLR, MLR, PLR, PIV, and Ki-67 index were determined as ≥ 2.34, ≥ 0.22, ≥ 131.8, ≥ 306.4, and ≥ 27, respectively.

**Table 2:** Receiver operating characteristic curve analyses for pathological complete response.

Curve	Cut-off value	AUC	95% CI	*p* value	Sensitivity (%)	Specificity (%)
NLR	2.34	0.552	0.494–0.609	0.105	74.7	38.2
MLR	0.22	0.604	0.542–0.667	0.001	63.6	54.2
PLR	131.8	0.567	0.507–0.626	0.036	69.7	44.8
PIV	306.4	0.592	0.539–0.645	0.004	81.8	45.8
Ki-67 index	27%	0.707	0.657–0.758	< 0.001	73.2	55.5

*AUC* the area under the curve, *CI* confidence interval, *NLR* neutrophil-to-lymphocyte ratio, *MLR* monocyte-to-lymphocyte ratio, *PLR* platelet-to-lymphocyte ratio, *PIV* pan-immune-inflammation-value.

### Outcomes

Table 3 depicts the analyses of the association between the patients' characteristics and pCR. The clinical tumor (T) stage, NLR, MLR, PLR, PIV, estrogen receptor (ER) status, human epidermal growth factor receptor-2 (HER-2) status, and Ki-67 index were significantly associated with response to NAC. However, multivariate logistic regression analysis revealed that the clinical T stage (odds ratio [OR], 2.16; 95% confidence interval [CI], 1.10–4.23; *p* = 0.025) , PIV (OR, 3.32; 95% CI, 1.53–7.21, *p* = 0.002), ER status (OR, 3.24; %95 CI, 1.84–5.71; *p* < 0.001), HER-2 status (OR, 3.84; 95% CI, 2.17–6.80; ip < 0.001), and Ki-67 index (OR, 3.30; 95% CI, 1.72–6.32; *p* < 0.001) were independent predictors for pCR (Table 4). The blood-derived inflammation markers other than PIV lost statistical significance in multivariate analysis.

**Table 3:** Association between the patients' characteristics and pCR.

Characteristic	pCR		*P* value
	Yes	No	
**Median age, years (minimum–maximum)**	49.5 (23.5–77.7)	47.8 (22.0–83.5)	0.694^a^
**Menopausal status, n (%)**			0.903^b^
*Premenopausal*	54 (50.5)	325 (51.1)	
*Postmenopausal*	53 (49.5)	311 (48.9)	
**Clinical T stage, n (%)**			**0.019**^*b*^
*T1/2*	86 (81.9)	432 (70.8)	
*T3/4*	19 (18.1)	178 (29.2)	
**Clinical Node status, n (%)**			0.681^b^
*Negative*	39 (42.0)	240 (39.0)	
*Positive*	67 (58.0)	377 (61.0)	
**NLR, n (%)**			**0.015**^b^
Low	74 (74.7)	308 (61.8)	
High	25 (25.3)	190 (38.2)	
**MLR, n (%)**			**0.001**^b^
Low	63 (63.6)	229 (46.0)	
High	36 (36.4)	269 (54.0)	
**PLR, n (%)**			**0.001**^*b*^
Low	72 (72.7)	275 (55.2)	
High	27 (27.3)	223 (44.8)	
**PIV, n (%)**			** < 0.001**^b^
Low	81 (81.8)	270 (54.2)	
High	18 (18.2)	228 (45.8)	
**ER status, n (%)**			**< 0.001**^b^
*Negative*	63 (60.6)	121 (21.5)	
*Positive*	41 (39.4)	443 (78.5)	
**HER-2 status, n (%)**			**< 0.001**^b^
*Negative*	55 (52.9)	453 (80.3)	
*Positive*	49 (47.1)	111 (19.7)	
**Ki-67 index, (%)**			**< 0.001**^b^
*Low*	17 (17.5)	230 (46.9)	
*High*	80 (82.5)	260 (53.1)	
**Histopathology, n (%)**			0.265^b^
*Invasive ductal carcinoma*	101 (95.3)	570 (92.2)	
*Other histology types*	5 (4.7)	48 (7.8)	
**Chemotherapy, n (%)**			0.423^b^
*Anthracycline plus taxane*	77 (72.0)	433 (68.1)	
*Other regimens*	30 (28.0)	203 (31.9)	

*pCR* pathological complete response, *NLR* neutrophil-to-lymphocyte ratio, *MLR* monocyte-to-lymphocyte ratio, *PLR* platelet-to-lymphocyte ratio, *PIV* Pan-immune-inflammation-value, *ER* estrogen receptor, *HER-2* human epidermal growth factor receptor-2.

^a^Mann-Whitney test; ^b^Pearson's Chi-squared test.

Data are given as counts (percentages) unless otherwise indicated.

Bold numbers indicate statistical significance.

**Table 4:** Univariate and multivariate logistic regression analysis for the predictors of pathological complete response.

Factor		Univariate Analysis				Multivariate Analysis			
		OR	95% CI		*P* value	OR	95% CI		*P* value
			Lower	Upper			Lower	Upper	
**Age**	*Years*	1.00	0.99	1.02	0.775				
**Menauposal status**	*Premen (RC) vs. postmen*	1.03	0.68	1.55	0.903				
**Clinical T stage**	*T3/4 (RC) vs. T1/2*	**1.87**	**1.10**	**3.16**	**0.020**	**2.16**	**1.10**	**4.23**	**0.025**
**Clinical Node status**	*Positive (RC) vs. negative*	0.91	0.60	1.40	0.681				
**NLR**	*High (RC) vs. low*	**1.83**	**1.12**	**2.98**	**0.016**	0.96	0.46	2.00	0.909
**MLR**	*High (RC) vs. low*	**2.06**	**1.32**	**3.21**	**0.002**	1.11	0.61	2.03	0.735
**PLR**	*High (RC) vs. low*	**2.16**	**1.34**	**3.48**	**0.002**	1.80	0.95	3.44	0.074
**PIV**	*High (RC) vs. low*	**3.80**	**2.21**	**6.52**	**< *0.001***	**3.32**	**1.53**	**7.21**	**0.002**
**ER status**	*Positive (RC) vs. negative*	**5.63**	**3.62**	**8.75**	**< *0.001***	**3.24**	**1.84**	**5.71**	**< *0.001***
**HER-2 status**	*Negative (RC) vs. positive*	**3.64**	**2.35**	**5.63**	**< *0.001***	**3.84**	**2.17**	**6.80**	**< *0.001***
**Ki-67 index**	*Low (RC) vs. high*	**4.16**	**2.40**	**7.24**	**< *0.001***	**3.30**	**1.72**	**6.32**	**< *0.001***
**Histopathology**	*IDC vs. other*	0.59	0.23	1.51	0.271				
**Chemotherapy**	*AplusT vs. other*	0.83	0.53	1.31	0.424				

*OR* odds ratio, *CI* confidential interval, *RC* reference category, *NLR* neutrophil-to-lymphocyte ratio, *MLR* monocyte-to-lymphocyte ratio, *PLR* platelet-to-lymphocyte ratio, *PIV* Pan-immune-inflammation-value, *ER* estrogen receptor, *HER-2* human epidermal growth factor receptor-2, *IDC* invasive ductal carcinoma, *AplusT* anthracycline plus taxane.

The multivariate logistic regression model is significant (*p* < 0.001).

Bold numbers indicate statistical significance.

**Figure 1:**
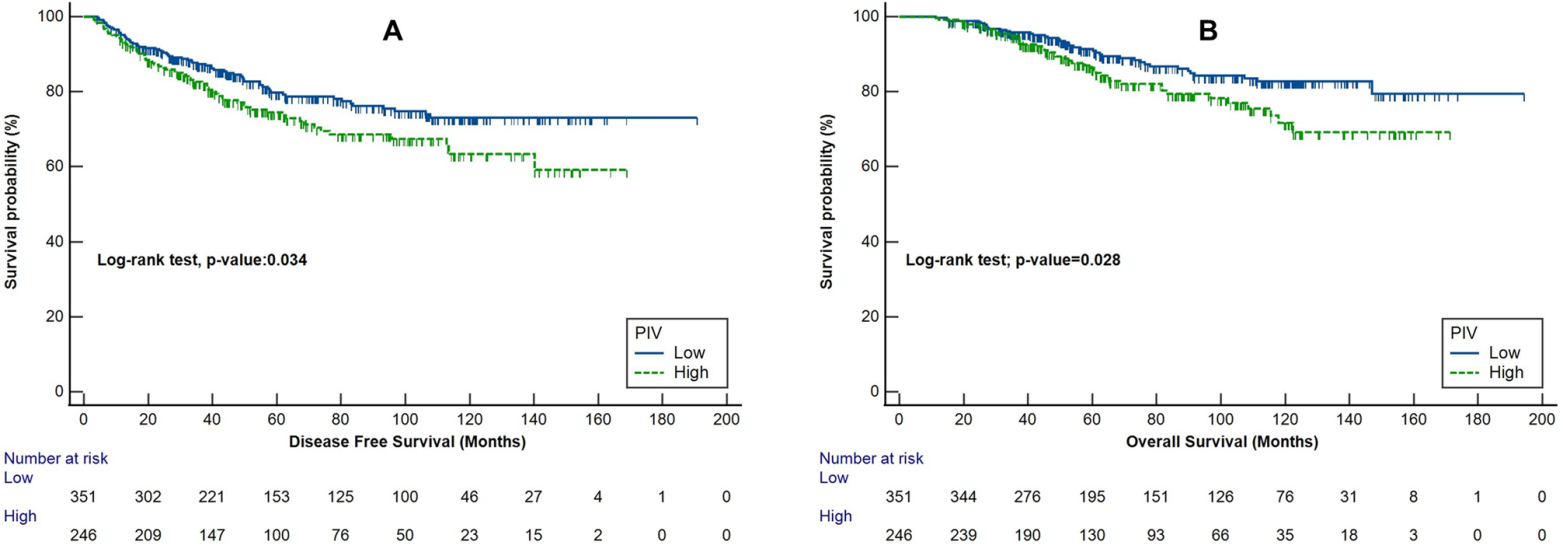
Kaplan-Meier plots of survival endpoints in different Pan-Immune-Inflammation-Value (PIV) groups (low *versus* high): (**A**) disease-free survival; (**B**) overall survival.

The median follow-up time was 67.5 months (range: 10.5–194.4 months). Median DFS and OS were not reached. 12-, 36-, and 60-months disease-free survival rates were 94.6%, 84.6%, 77.5%, respectively. 12-, 36-, and 60-months overall survival rates were 99.6%, 95.4%, 88.7%, respectively. As a secondary outcome, the disease-free survival (DFS) of patients in the low PIV group was significantly longer than the DFS of the high PIV group patients (hazard ratio [HR], 0.69; 95% CI 0.49–0.97; *p* = 0.034; Fig. 1A). Similarly, the low PIV group patients had significantly better overall survival (OS) than those in the high PIV group (HR, 0.61; 95% CI 0.39–0.95; *p* = 0.028; Fig. 1B).

## Discussion

In the present study, we demonstrated for the first time that a new inflammatory score, PIV, was one of the independent predictors for pCR to NAC like the well-studied other clinicopathological factors such as T stage, ER status, HER-2 status, and Ki-67 index in breast cancer. Additionally, PIV outperformed other blood-derived inflammation markers in predicting pCR, and it also had a prognostic value for DFS and OS.

In general, white blood cell count reflects an individual's systemic and/or local inflammatory status^11^. Neutrophils are known to regulate the tumor microenvironment and produce cytokines, chemokines, and growth factors that may promote angiogenesis as well as tumor cell proliferation and migration^19^. The M2 phenotype tumor-associated macrophages (TAMs) deriving from circulating monocytes exist within the tumor microenvironment and promote metastasis and immunosuppression^20,21^. It was reported that peripheral monocyte count was associated with the density of the TAMs, and high absolute monocyte count predicted poor survival in cancer patients^22,23^. Platelets are other cells contributing to cancer-favored inflammation by various mechanisms. For example, the activated platelets inhibit the interaction between tumor cells and cytolytic immune cells by forming a layer around tumor cells and support tumor growth via the secretion of several factors such as TGF-β^24,25^. Hence, high platelet counts were reported to be associated with adverse outcomes in breast cancer^26^. In contrast, lymphocytes are responsible for antitumor-specific immune response—including T-lymphocyte tumor infiltration and cytotoxic T-lymphocyte-mediated antitumor activity^11,27^. Starting from these findings, NLR, MLR, and PLR, indexes reflecting the balance between inflammation and immunoreaction in cancer, were reported to have predictive value in NAC response in many breast cancer studies, supporting our findings of univariate analysis^15–17,28^.

Although the scientific evidence supporting the predictive value of the blood-derived inflammation indexes has been expanding, there are conflicting reports about which index provides the best prediction for the efficacy of NAC in breast cancer^29–33^. For example, Eren et al. reported that NLR was the only independent predictive factor of pCR among blood-derived inflammation markers in multivariate analysis^29^. In another study conducted by Peng et al., multivariate analysis of 808 breast cancer patients showed that the lymphocyte-monocyte ratio was the only independent predictive factor for the efficacy of NAC among these inflammatory markers^32^. In addition, Hu et al. stated that PLR had superior efficacy to NLR in predicting NAC response^33^. When these studies are evaluated together with scholars presenting negative results^34^, it has emerged offering a combination of these markers^35,36^. The combination of NLR and PLR was reported to predict NAC response more precisely than NLR and PLR alone, claiming that the combination of different biomarkers could better define the patients' inflammatory status^35,36^.

PIV is a new blood-based biomarker integrating different peripheral blood immune cell subpopulations-neutrophil, platelet, monocyte, and lymphocyte. Due to its potential to represent comprehensively patient's immunity and systemic inflammation, PIV was proposed as a stronger predictor of outcomes in advanced cancer patients receiving cytotoxic chemotherapy, immunotherapy, and targeted therapy^18,37–39^. Recently, Ligorio reported that PIV was firmly associated with survival and outperformed NLR, PLR, and MLR in predicting survival in patients with HER-2 positive advanced breast cancer^39^. In line with the studies mentioned above, we showed that patients with low PIV scores had better survival outcomes. Furthermore, to our knowledge, this is the first study reporting that PIV was a more reliable predictor of pCR after NAC than other blood-based markers in breast cancer patients.

Meta-analyses and systematic reviews have shown that numerous factors—including age, genetic polymorphisms, tumor-infiltrating lymphocytes, programmed death-ligand 1, ER, progesterone receptor, and HER2 expression status—may be predictive of response to NAC in women with breast cancer^7,40–42^. However, most of these variables become available only following detailed pathological and genetic investigations. There is, therefore, an urgent need for reliable prognostic tools grounded on simple pre-treatment variables. In this scenario, PIV—an easy-to-drive biomarker originating from routine complete blood count—may help clinicians to predict treatment responses after prospective validation and confirmation of our results by further studies.

Some limitations of our study merit comment, including the retrospective design, the presentation of single-center experience, and the inclusion of women of Turkish descent only. Furthermore, although we excluded the patients with hematological disorders and those receiving immunomodulatory treatment, various other conditions may influence the blood-based biomarkers.

Based on our results, pre-treatment PIV seems to have a significant predictive value and outperform NLR, MLR, PLR in predicting pCR in Turkish women with breast cancer who received NAC. In addition, PIV has a prognostic impact on survival. However, further studies are needed to confirm our findings.

## Materials and methods

### Study population

**Figure 2:**
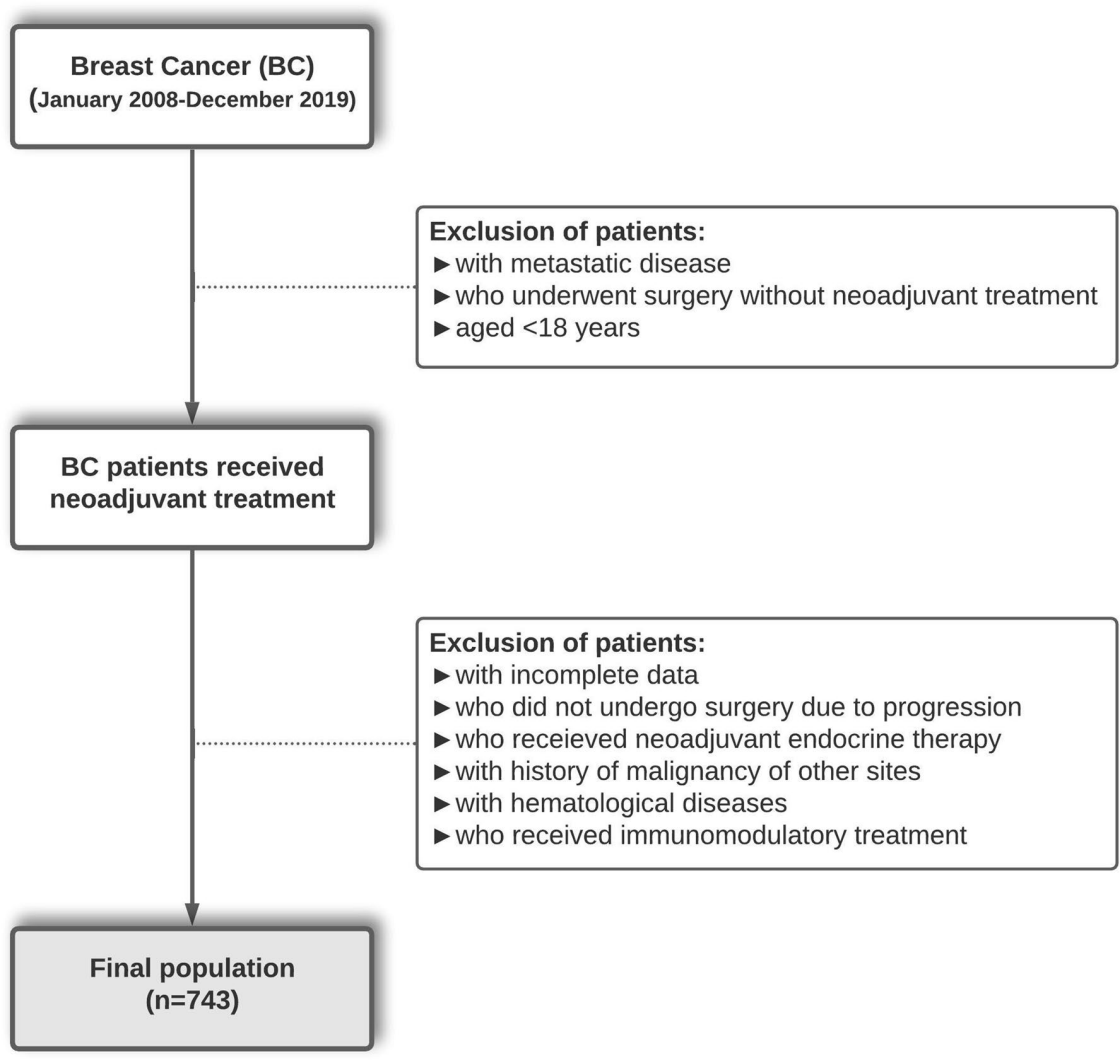
Flowchart of patients selection.

Figure 2 shows the profile of our study. The electronic records of patients admitted to the Department of Oncology or the Department of General Surgery, Uludag University Medical Center (Bursa, Turkey) between January 2008 and December 2019 due to breast cancer were reviewed. Among patients who underwent NAC before attempting cytoreductive surgery, patients who were aged < 18 years, received immunomodulatory treatment or neoadjuvant endocrine therapy, had a history of malignancies at other sites, hematological disease, incomplete data were excluded. The study consisted of 743 Turkish women.

### Data collection

The following variables were extracted from medical records in all participants: age; menopausal status; pre-treatment T stage; pre-treatment axillary lymph node status; pre-treatment histotype; pre-treatment expression of ER, HER-2, and Ki-67; and chemotherapy regimens. By immunohistochemical staining, ER expression levels ≥ 1% were considered positive. The final pathological reports of all patients were reviewed for pCR. According to the American Joint Committee on Cancer (AJCC) Staging Manual, eighth edition, the staging of the patients was carried out. A pre-treatment complete blood count was obtained in the two weeks preceding NAC. The absolute counts of neutrophils, monocytes, platelets, and lymphocytes were used to estimate NLR, MLR, and PLR. The PIV was calculated by multiplying neutrophil count (10^3^/mL) by platelet count (10^3^/mL) and monocyte count (10^3^/mL) and dividing the result by lymphocyte count (10^3^/mL).

### Ethical statement

The study protocol complied with the tenets of the Helsinki declaration, and ethical approval was granted by the Institutional Review Board of Bursa Uludag University (Approval Number: 2020-6/31). The Clinical Research Ethics Committee of the Bursa Uludag University Faculty of Medicine waived the need for informed consent due to the study's retrospective nature.

### Outcomes

The primary outcome measure was pCR to NAC. The pCR was defined as the absence of tumor cells in both the mastectomy specimen and the sampled or dissected regional lymph nodes. Secondary outcome measures included DFS and OS. DFS was calculated as the time (in months) from curative surgery until recurrence or death, whichever occurred first. OS was calculated as the time (in months) from breast cancer diagnosis to death.

### Statistical analysis

The optimal cut-off points for NLR, MLR, PLR, PIV, and Ki-67 index were determined using ROC curve analysis, taking pCR as the endpoint of interest. The general characteristics of the study patients are presented using descriptive statistics (median, ranges, counts, and percentages). The Pearson's Chi-squared test (categorical variables) or the Mann–Whitney U test (continuous variables) were used to analyze the association between pCR and the variables. Binary logistic regression analysis was employed for multivariate analysis, including the factors having a p-value below 0.25 in univariate analysis. Survival curves were plotted using the Kaplan–Meier method and compared with the log-rank test. All calculations were performed using SPSS, version 22.0 (IBM, Armonk, NY, USA) and MedCalc Statistical Software trial version 20.009 (MedCalc Software bv, Ostend, Belgium; www.medcalc.org; 2021). Two-tailed *p* values < 0.05 were considered statistically significant.
